# Trends in psychiatric diagnoses, medications and psychological therapies in a large Swedish region: a population-based study

**DOI:** 10.1186/s12888-020-02749-z

**Published:** 2020-06-23

**Authors:** T. Forslund, K. Kosidou, S. Wicks, C. Dalman

**Affiliations:** 1 Department of Medicine Solna, Centre for Pharmacoepidemiology, Karolinska Institutet, Stockholm, Sweden; 2 Public Healthcare Services Committee, Department of Healthcare Development, Box 6909, 102 39 Stockholm, Stockholm Region Sweden; 3 Department of Public Health Sciences, Karolinska Institutet, Stockholm, Sweden; 4 Centre for Epidemiology and Community Medicine, Stockholm, Stockholm Region Sweden

## Abstract

**Background:**

Health services utilization for mental health disorders is reported to increase sharply in many countries. The aim of this study was to report trends in all aspects of mental health care utilization in a total population sample.

**Methods:**

Repeated cross-sectional register study of the Stockholm Region (VAL) including both primary and secondary care. Trends in the proportion of adults in the total population of Stockholm Region with a recorded ICD-10 psychiatric diagnosis or psychological therapy during 2007–2017 as well as claims of psychiatric medication from 2011 were calculated.

**Results:**

The proportion of adults utilizing any mental health care increased from 13.2% in 2011 to 16.1% in 2017. In 2017, 49.3% were treated in primary care, 32.2% in secondary care and 18.5% were jointly managed. The increase was most pronounced in younger adults. Women were more likely to receive mental health care than men in all ages. Medication decreased from 71.0 to 67.7%, while psychological therapy increased from 33.1 to 37.6%. The use of psychiatric medication increased with age while psychological therapy decreased. All time trends were statistically significant (*p* < .0001).

**Conclusion:**

Care for mental health disorders has been increasing mainly in primary care and was delivered to one in seven adult individuals in 2017. Interventions are needed to address the growing burden of mental health disorders while avoiding overtreatment.

## Background

Mental disorders contribute significantly to the global disease burden [1]. It has been estimated that 35% of all adults in developed countries will develop a clinically manifest mental disorder during their lifetime [2]. Mental disorders tend to be chronic or relapse; and they have serious consequences on the individual and societal level [1]. Help seeking appears to increase, especially among young adults [1, 3–9], although no study has yet reported on both primary and secondary care with individual level data over time.

There is no compelling evidence of increases in the actual psychiatric morbidity in developed countries. For example, population-based studies using structured diagnostic interviews repeated over time report stable levels of clinically significant depression and anxiety [10, 11]. However, many studies from Sweden and other developed countries have shown increasing long-term trends in self-reported depressive and anxiety symptoms, particularly among young people [5, 6, 12–16] Taken together, these findings may suggest an increase in psychiatric symptoms over time while more severe psychiatric morbidity remains stable.

Increased awareness about mental health disorders and extensive anti-stigma campaigns may have enhanced help seeking among adults and children [17–20]. A recent Swedish study found a lower threshold for seeking mental health care among young adults during recent years, however still at an equal level as the older age groups, indicating decreasing unmet needs among the young [21].

During the past three decades there have been major shifts in care for mental health disorders. The introduction and widespread use of selective serotonine uptake inhibitors (SSRI) [22, 23] and psychological therapy including cognitive behavioral therapy (CBT) [24, 25] meant that well tolerated and effective treatments for depression and anxiety disorders became available in primary care, widening the group of individuals eligible for treatment. Further, the use of psychostimulants and the increased interest in Attention-deficit/ hyperactivity disorder has led to increased access to treatment for large numbers of children and adults [26].

Efforts have been made to increase the accessibility of services for mental health care in many countries [17, 27]. To reduce potential inequalities in care and improve care provision there has been an explicit call to quantify changes in mental health services utilization and treated prevalence of mental disorders across countries [27].

In summary, the utilization of care for mental health disorders is increasing, probably due to a combination of reasons. In order to design and implement interventions targeted at optimizing care for mental health disorders several questions remain to be clarified. A study describing all aspects of mental health care in both primary and secondary care in a total population sample is missing and could give further insights into how to plan the provision of mental health care in the future.

The aims of this study were:

1. To describe trends in the proportion of adults that utilize overall mental health care each year in the entire adult population of the Stockholm Region during the years 2011–2017, covering both primary and secondary care
2. To describe the content of overall care defined as the proportion of adults in Stockholm Region with prescription claims of psychiatric medication, psychological therapies and registered psychiatric diagnoses over time in both primary and secondary care, and their overlap at the individual level.
3. To examine sex- and age differences in mental health care utilization

## Method

A repeated population based cross-sectional study of the total adult population in the Stockholm Region was undertaken using administrative health care data from 2007 to 2017. The Stockholm Region has had an increasing population from 1.47 million adult inhabitants in 2007 to 1.76 million adult inhabitants in 2017. Psychiatric care in the Stockholm Region is delivered by 15 hospitals providing inpatient care, 14 specialist outpatient care centers of varying size and number of subspecialized units, and approximately 200 primary care centers. The primary care centers employ physicians, nurses, nursing-aids; but also counselors, psychologists, and psychotherapists who provide both mental health counseling and psychotherapy.

Data was extracted from the administrative health care data register VAL (Vårdanalysdatabaserna; Stockholm regional health care data warehouse). It contains pseudonymized individual level data regarding diagnoses, age, sex, prescription claims, psychological therapies, hospitalizations and other healthcare consultations within all publicly funded health care, as well as migration and death for all individuals in the region (1.76 million adult inhabitants). VAL also contains individual level data on all prescription drugs (ATC-codes: Anatomic Therapeutic Chemical classification system) dispensed anywhere in Sweden to inhabitants in the region since July 2010. This information is derived from the national prescribed drug register [28].

Secondary health care consultations and hospitalizations are identical to the National Patient Register (NPR) which has previously been extensively used in epidemiological research and proved to be of sufficient quality and coverage [29]. However, VAL has the advantage of also including primary care.

### Psychiatric diagnoses, prescription claims of psychiatric medication, and psychological therapies

The definitions of psychiatric diagnoses and psychiatric medication can be found in Supplemental Tables 1 and 2. The burnout syndrome has been defined as an ICD-10 diagnosis (International Statistical Classification of Diseases and Related Health Problems - Tenth Revision) by the Swedish National Board of Health and Welfare and includes criteria of exhaustion and lack of mental energy caused by long-term stress. Amitriptylin (N06AA09) was excluded from the analyses since it is mainly used for long term pain. Psychological therapy was defined as counseling (registered physical visit) with either a counselor, psychologist or psychotherapist within the primary health care system or within the secondary specialized psychiatric care during 2007–2017.

### Definition of mental health care utilization

An individual was considered as having received any mental health care when she or he had a registered psychiatric diagnosis according to Supplemental Table 1, claimed a prescribed psychiatric medication according to Supplemental Table 2 or having received psychological therapy from 2011 to 2017. This definition was based on previous work by our research group [30].

### Statistics

Descriptive statistics were used for crude estimates with data presented as proportions or relative risks with 95% confidence intervals (CI), as appropriate. The Cochran–Armitage test was used for trend statistics. Direct standardization was used to calculate age and sex specific trends in the proportions of adults with registered psychiatric diagnoses, prescription claims of psychiatric medication and receipt of psychological therapy each year during 2011–2017. Trends in mental health care were also calculated by level of health care, including primary and secondary care. The proportion of individuals with psychiatric diagnoses in different levels of care during a 5-year period as well as overlaps between recorded diagnoses, psychiatric medication and receipt of psychological therapy were described. The proportion of individuals with at least two diagnoses during a five-year period was calculated. Age by December in the year of analysis was used for stratifications and adjustments. Four age groups were used: 20–29, 30–44, 45–64, and 65+ years.

## Results

### Time trends in the overall mental health care utilization

**Fig. 1:**
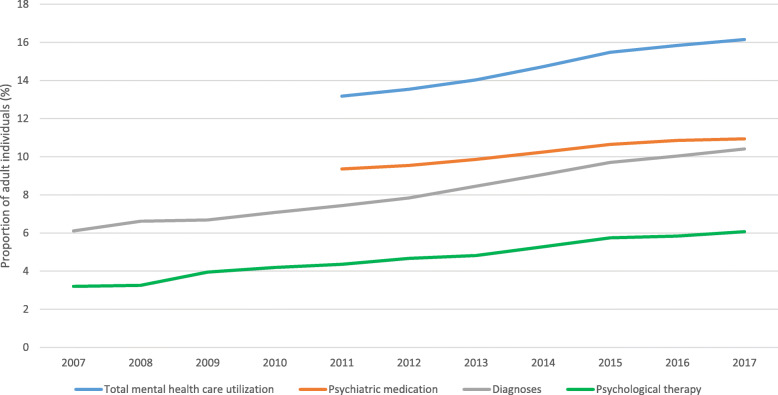
Age and sex standardized time trends in registered psychiatric diagnosis, prescription claims of psychiatric medication and/or receipt of psychological therapy among adults in the Stockholm Region (1,758,337 adult individuals in 2017)

The proportion of adults in the total population of the Stockholm Region that received mental health care increased steadily each year between 2011 and 2017 (Table 1, Fig. 1). Time trends for all subgroups were statistically significant (*p* < .0001) (Table 1). In the year 2017, 16.1% of adults in the Stockholm Region received some type of mental health care. All components of care including registered psychiatric diagnosis, prescription claims of psychiatric medication and/or receipt of psychological therapy increased during the study period (Fig. 1). Among the individuals who utilized mental health care, use of medication decreased from 71.0 to 67.7%, while psychological therapy increased from 33.1 to 37.6% (data not shown).

**Table 1:** Mental health care utilization including psychiatric diagnoses, psychiatric medication and psychological therapy per year in adult individuals in the Stockholm Region

	2007	2008	2009	2010	2011	2012	2013	2014	2015	2016	2017	Relative Risk 2017 vs. 2011	Trend
Adult Population	*n* = 1,472,348	*n* = 1,496,556	*n* = 1,526,414	*n* = 1,554,739	*n* = 1,585,922	*n* = 1,613,526	*n* = 1,642,837	*n* = 1,671,651	*n* = 1,695,318	*n* = 1,722,808	*n* = 1,758,337		
	n (%)	n (%)	n (%)	n (%)	n (%)	n (%)	n (%)	n (%)	n (%)	n (%)	n (%)	RR (CI)	p
**Any mental health care**	–	–	–	–	208,977 (13.2%)	218,454 (13.5%)	230,456 (14.0%)	246,178 (14.7%)	262,396 (15.5%)	272,841 (15.8%)	283,910 (16.1%)	1.22 (1.22–1.23)	< .0001
**Psychiatric diagnoses**	89,994 (6.1%)	99,083 (6.6%)	102,008 (6.7%)	110,034 (7.1%)	117,948 (7.4%)	126,451 (7.8%)	138,947 (8.5%)	151,656 (9.1%)	164,461 (9.7%)	172,961 (10.0%)	183,104 (10.4%)	1.40 (1.39–1.41)	< .0001
*Anxiety disorders*	29,410 (2.0%)	34,914 (2.3%)	38,172 (2.5%)	41,916 (2.7%)	45,266 (2.9%)	49,988 (3.1%)	58,438 (3.6%)	66,476 (4.0%)	73,559 (4.3%)	78,773 (4.6%)	86,310 (4.9%)	1.72 (1.71–1.74)	< .0001
*Depression*	42,931 (2.9%)	46,418 (3.1%)	47,061 (3.1%)	50,108 (3.2%)	52,529 (3.3%)	55,584 (3.4%)	60,247 (3.7%)	62,715 (3.8%)	65,727 (3.9%)	66,850 (3.9%)	67,696 (3.8%)	1.16 (1.15–1.18)	< .0001
*Stress/Adjustment disorders*	19,823 (1.3%)	21,680 (1.4%)	21,615 (1.4%)	22,785 (1.5%)	24,494 (1.5%)	27,123 (1.7%)	29,910 (1.8%)	34,716 (2.1%)	41,302 (2.4%)	45,264 (2.6%)	47,730 (2.7%)	1.76 (1.73–1.78)	< .0001
*ADHD*	1166 (0.1%)	2136 (0.1%)	3286 (0.2%)	5207 (0.3%)	7275 (0.5%)	9545 (0.6%)	11,849 (0.7%)	14,485 (0.9%)	16,742 (1.0%)	18,877 (1.1%)	21,275 (1.2%)	2.64 (2.57–2.71)	< .0001
*Burnout syndrome*	408 (0.0%)	893 (0.1%)	1397 (0.1%)	2003 (0.1%)	2782 (0.2%)	3646 (0.2%)	5244 (0.3%)	7733 (0.5%)	11,433 (0.7%)	14,968 (0.9%)	17,264 (1.0%)	5.60 (5.38–5.83)	< .0001
*Bipolar disorders*	4050 (0.3%)	4914 (0.3%)	5344 (0.4%)	6015 (0.4%)	6527 (0.4%)	6956 (0.4%)	7843 (0.5%)	8491 (0.5%)	8976 (0.5%)	9445 (0.5%)	9929 (0.6%)	1.37 (1.33–1.42)	< .0001
*Psychotic disorders*	7820 (0.5%)	8299 (0.6%)	8085 (0.5%)	8309 (0.5%)	8761 (0.6%)	9014 (0.6%)	9311 (0.6%)	9379 (0.6%)	9504 (0.6%)	9745 (0.6%)	9693 (0.6%)	1.00 (0.97–1.03)	< .0001
*Developmental disorders*	857 (0.1%)	1226 (0.1%)	1484 (0.1%)	2122 (0.1%)	2936 (0.2%)	3644 (0.2%)	4568 (0.3%)	5413 (0.3%)	6312 (0.4%)	7089 (0.4%)	8158 (0.5%)	2.51 (2.40–2.61)	< .0001
*Eating disorders*	1172 (0.1%)	1305 (0.1%)	1260 (0.1%)	1333 (0.1%)	1698 (0.1%)	1982 (0.1%)	2411 (0.1%)	2616 (0.2%)	2652 (0.2%)	2677 (0.2%)	2641 (0.2%)	1.40 (1.32–1.49)	< .0001
**Psychiatric medication**	–	–	–	–	148,374 (9.4%)	153,988 (9.5%)	161,971 (9.9%)	171,294 (10.2%)	180,463 (10.6%)	187,143 (10.9%)	192,342 (10.9%)	1.17 (1.16–1.18)	< .0001
*Antidepressants*	–	–	–	–	130,612 (8.2%)	135,466 (8.4%)	142,566 (8.7%)	150,202 (9.0%)	158,355 (9.3%)	163,938 (9.5%)	167,765 (9.5%)	1.16 (1.15–1.17)	< .0001
*Antipsychotics*	–	–	–	–	28,286 (1.8%)	28,646 (1.8%)	29,922 (1.8%)	31,628 (1.9%)	32,409 (1.9%)	33,206 (1.9%)	33,864 (1.9%)	1.08 (1.06–1.10)	< .0001
*Psychostimulants*	–	–	–	–	8009 (0.5%)	9591 (0.6%)	11,122 (0.7%)	12,975 (0.8%)	14,627 (0.9%)	16,258 (0.9%)	17,889 (1.0%)	2.01 (1.96–2.07)	< .0001
**Psychological therapy**	47,108 (3.2%)	48,746 (3.3%)	60,333 (4.0%)	65,167 (4.2%)	69,096 (4.4%)	75,363 (4.7%)	79,105 (4.8%)	88,265 (5.3%)	97,416 (5.7%)	100,536 (5.8%)	106,666 (6.1%)	1.39 (1.38–1.41)	< .0001

In the beginning of the study period (2011) the proportions of patients in primary and secondary care were similar (Supplemental Figure 1). By the end of the observation period (2017) most of the patients were completely (49.3%) or partly (18.5%) managed in primary care, whereas 32.2% were completely managed within secondary care (Supplemental Figure 1). The increase of health care utilization was exclusively found in patients treated in primary care (+ 36.1%) or in collaboration with secondary care (+ 41.0%), while isolated secondary care remained stable (− 0.2%) (data not shown). Of those with psychological therapy in 2017, 37.9% received it from secondary care only while 56.5% received it from primary care only, the corresponding figures for psychiatric medication were 43.8 and 46.1% for secondary care and primary care respectively (data not shown).

### Overlap between psychiatric diagnoses, psychiatric medication and psychological therapy

**Fig. 2:**
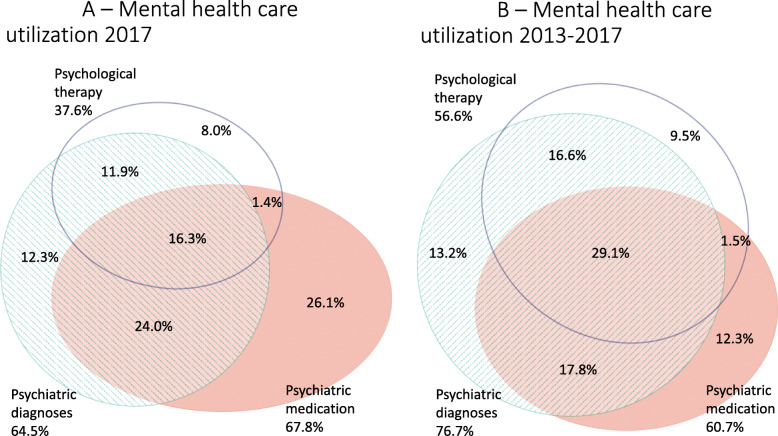
**a** and **b** Relationship between mental health care utilization measures in the Stockholm Region. 2A) During 2017 (*n* = 283,910; 16.1% of the adult population). 2B) During 2013–2017 (*n* = 470,779; 26.8% of the adult population)

Recorded diagnoses identified 65% of all the individuals that received care during 1 year (2017): 27.5% used psychiatric medication without a recorded diagnosis, while 9.4% received psychological therapy without a recorded diagnosis (Fig. 2a). When assessed during a 5-year period (2013–2017) the lack of overlap decreased (Fig. 2b).

### Prevalence of diagnosed psychiatric disorders and psychiatric medication

**Fig. 3:**
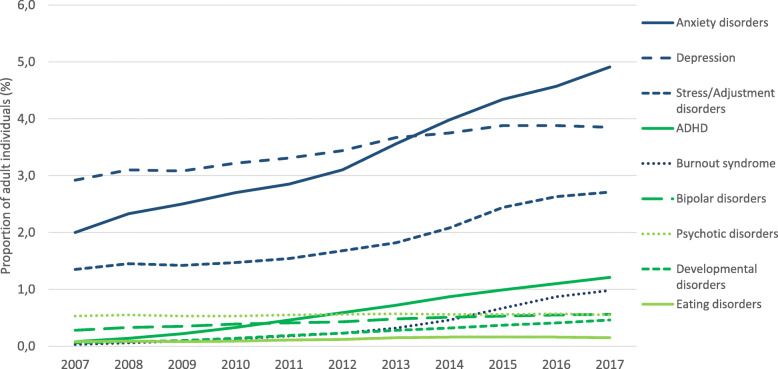
Age and sex standardized one-year prevalence of psychiatric disorders in adult individuals in the Stockholm Region 2007–2017 (1,758,337 individuals in 2017)

Figure 3 shows trends in the one-year prevalence of diagnosed psychiatric disorders in adults in Stockholm Region between 2007 and 2017 as measured by registered ICD-10 diagnoses. The most common disorders were depression, anxiety and stress/adjustment disorders, which all increased during the study period. At end of the observation window 4.9% of adults in the Stockholm Region had a diagnosis of anxiety and 3.8% had depression. Large increases in diagnoses for burnout syndrome, ADHD (Attention-Deficit/Hyperactivity Disorder), and developmental disorders were observed during the study period. By contrast, psychotic disorders remained stable (Table 1, Fig. 3).

Correspondingly, the proportion of adults prescribed antidepressants and psychostimulants increased between 2011 and 2017, while use of antipsychotic drugs remained stable (Table 1, Supplemental Figure 2).

Individuals with diagnosed depression, anxiety or stress disorders were more commonly found within primary than secondary care. By contrast, individuals with diagnosed ADHD, psychotic disorders, bipolar disorders and eating disorders were mainly found in secondary care (Supplemental Figure 3). There is substantial overlap between the diagnoses during the 5- year period: 65% of the individuals are included in at least two of the groups (data not shown).

### Sex- and age differences in mental health care utilization

**Fig. 4:**
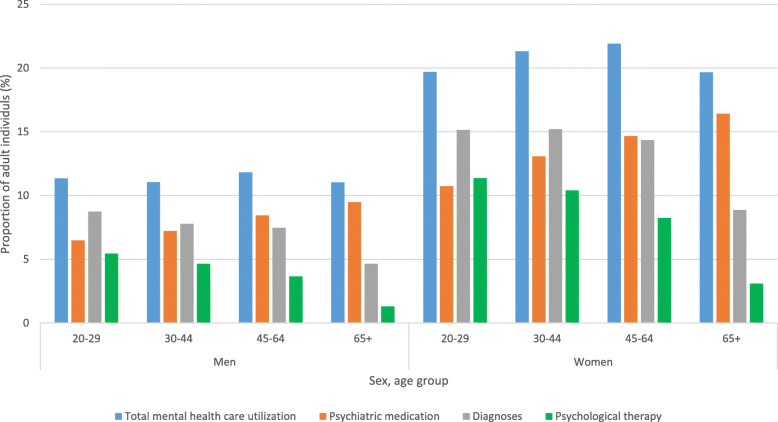
Proportion of adults in the total population of Stockholm Region receiving mental health care in the year 2017, including a registered psychiatric diagnosis, prescription claims of psychiatric medication, and/or receipt of psychological therapy by sex and age group (Stockholm Region population 1,758,337 individuals)

Overall, in 2017 women were 1.5–2 times more likely than men to utilize mental health care in all age groups (20–29 years: RR (relative risk) 1.66 (1.62–1.70); 30–44 years: RR 1.93 (1.90–1.95); 45–64 years: RR 1.85 (RR 1.83–1.88); 65+ years: RR 1.78 (1.75–1.81)) (Fig. 4). The difference was most pronounced for women regarding utilization of psychological therapy (20–29 years: RR 2.09 (2.04–2.14)); 30–44 years: RR 2.24 (2.19–2.29); 45–64 years: RR 2.25 (2.20–2.30); 65+ years: RR 2.35 (2.24–2.47)). The sex difference in utilization of mental health care was more pronounced in the primary care setting (RR women vs. men 2.12 (2.09–2.14). The difference was smaller in the secondary care setting (RR women vs. men 1.37 (1.36–1.39). The gender gap in the utilization of mental health care remained stable throughout the study period.

Among adult women, mental health care utilization peaked in the 45–64 age group with a prevalence of 21.9% in 2017 (Fig. 4). Among men, there was little variation in the overall mental health care utilization by age group. Regardless of gender, the use of psychiatric medication increased with age while psychological therapy decreased with age. Recorded diagnoses were less commonly found in the oldest patients over the age of 65 (Fig. 4). Stratified analyses reveal that the increase in mental health care utilization was most pronounced in younger individuals but decreased with age in both men and women (Supplemental Figure 4 and 5). At end of the observation window younger adults utilized mental health care at almost the same level as those of middle-age.

## Discussion

In this, to our knowledge first, total population study of time trends between 2011 and 2017 in all aspects of mental health care utilization including both primary and secondary care, we found that a steadily increasing proportion of adults are utilizing mental health care, reaching 16.1% of all adults in the Stockholm Region in 2017. The proportion of the individuals in care receiving medication decreased slightly, while psychological therapy increased. Most individuals utilizing mental health care were diagnosed with depression, anxiety or stress/adjustment disorders. Almost twice as many women than men utilized mental health care in all age groups and the gender gap remained stable during the study period. Women utilized both more primary and secondary mental health care than men, but the difference was larger for primary care utilization. Health care utilization increased in all age groups but was more pronounced in younger individuals resulting in similar levels of mental health care utilization across age groups in 2017. The use of psychiatric medication increased with age while the opposite was true for psychological therapy. During 2017 medication and psychological therapy were only partly overlapping, while psychiatric diagnoses often were missing. However, a 5-year time window revealed increasing overlaps indicating long term efforts to treat specific mental health disorders using several treatment options.

Isolated evidence from various parts of the health care suggests that help-seeking for mental health problems has increased in many developed countries [17–20, 31, 32]. Our findings using total population data demonstrate an increase in the total mental health care utilization mainly explained by an increased use of primary care and by younger adults.

We found evidence of an increase in diagnoses of almost all mental disorders except psychotic disorders. Although our findings are by no means evidence of a true increase in the prevalence of these mental disorders, such an increase cannot be ruled out.

Finally, our study found a high use of psychiatric medication relative to psychiatric diagnoses in individuals above the age of 65 which might reflect off-label use of medication or physicians being less inclined of registering all diagnoses in individuals with multimorbidity.

### Strengths

The use of data from high quality registers that cover the total population of the Stockholm Region and include all publicly funded health care is a major strength of our study. Our individual level data allowed us to examine overlaps in the content of care over a long period of time. This total population perspective increases the validity of our findings in comparison to studies from other healthcare systems and regions where such information is lacking.

### Limitations

There were some limitations of the study. We had no information on the type of psychological therapy. The estimate of mental health care utilization is conservative and neither included psychological distress; nor insomnia, medications intended for sleep disorders, or short-term treatment of anxiety. Privately funded psychological therapy or therapy within the work place environment could not be included as it is not recorded in VAL. However, given our knowledge of the health care system we believe that most of these patients will utilize publicly funded health care as well. Also, all prescription claims are covered in the VAL-database. Alcohol and substance use disorders were not studied, but are underreported in health care registries, and might warrant other methods and data sources than the present study. In 2013 there was a change from the simplified ICD-10P to ICD-10 in primary care which might have affected registration routines.

### Generalizability

Access to health care is better in the Stockholm Region than in other less urbanized parts of the country. We believe the region to be representative of other urbanized regions in Sweden (comprising approximately 60% of the Swedish population) and of urbanized areas of other European countries with publicly funded health care.

### Implications

This study clearly shows that mental health disorders are an important and increasing part of modern health care. At end of the observation window the majority of individuals with mental health disorders were managed in primary care or in collaboration with secondary care with access to psychological therapy.

A Swedish screening study with data from 2009 reported point prevalence of clinically relevant depression or anxiety of 17.2% [33]. Since 16% of the adult population utilized mental health care in 2017 one may speculate that the increasing number of individuals seeking care will continue as there are obviously other diagnoses than anxiety and depression. The overall trends described raise the question about what to do and how to best take care of all the people in need of support. Apart from traditional specialized care and primary care there might be a need to develop other types of support functions, and to structure preventive work, in other parts of society than the health care system.

### Further research

The overlap over time between diagnoses and given treatments on an individual level should be further investigated. The lack of diagnoses among older individuals treated with psychiatric medication should be further explored. Another important future question is whether all the increasing efforts described above lead to clinically relevant improvements or if they simply substitute other types of social support? What is the content and quality of delivered care? Interventions for improving care without overtreating the steadily growing group of individuals with psychiatric disorders are needed.

## Conclusion

Care for mental health disorders has been increasing mainly in primary care and was delivered to one in seven adult individuals in 2017. The increase was most pronounced in younger adults whom at end of the observation window utilized mental health care at almost the same level as middle-aged. The proportion of individuals of those in care receiving medication decreased slightly, while psychological therapy increased from 2011 to 2017. The content of psychological therapy and the quality of care remain unknown. Interventions are needed to address the growing burden of mental health disorders while avoiding overtreatment.

## Supplementary information

**Additional file 1: Table S1.** ICD-code definitions. **Table S2.** ATC-code definitions. **Figure S1.** Age and sex standardized time trends for mental health care utilization in primary and secondary care respectively or both in the Stockholm Region (1,758,337 adult individuals in 2017). **Figure S2.** Age and sex standardized prescription claims of psychiatric medication (antidepressants, antipsychotics and psychostimulants) among adults in the Stockholm Region 2011–2017 (1,758,337 adult individuals in 2017). **Figure S3.** Adult individuals with a diagnosis of a psychiatric disorder during 2013–2017 by level of care in the Stockholm Region (population 1,758,337 individuals). **Figure S4.** Time trends in registered psychiatric diagnosis, prescription claims of psychiatric medication and/or receipt of psychological therapy among women in the Stockholm Region 2007–2017 per age group. **Figure S5.** Time trends in registered psychiatric diagnosis, prescription claims of psychiatric medication and/or receipt of psychological therapy among men in the Stockholm Region 2007–2017 per age group.

## Data Availability

The datasets generated during and/or analysed during the current study are not publicly available due to privacy restrictions but are available from the corresponding author on reasonable request.

## Abbreviations

ADHD: Attention-Deficit/Hyperactivity Disorder
ATC: Anatomic Therapeutic Chemical classification system
CI: 95% confidence intervals
ICD-10: International Statistical Classification of Diseases and Related Health Problems - Tenth Revision
NPR: National patient register
RR: Relative risk
VAL: Vårdanalysdatabaserna; Stockholm regional health care data warehouse
