# Supplementary material for: Trends in psychiatric diagnoses, medications and psychological therapies in a large Swedish region: a population-based study
Source: BMC Psychiatry. 2020 Jun 23;20:328. doi: 10.1186/s12888-020-02749-z (PMC7313191; doi:10.1186/s12888-020-02749-z)
Supplement: Supplementary file 1 — Additional file 1: Table S1. ICD-code definitions. Table S2. ATC-code definitions. Figure S1. Age and sex standardized time trends for mental health care utilization in primary and secondary care respectively or both in the Stockholm Region (1,758,337 adult individuals in 2017). Figure S2. Age and sex standardized prescription claims of psychiatric medication (antidepressants, antipsychotics and psychostimulants) among adults in the Stockholm Region 2011–2017 (1,758,337 adult individuals in 2017). Figure S3. Adult individuals with a diagnosis of a psychiatric disorder during 2013–2017 by level of care in the Stockholm Region (population 1,758,337 individuals). Figure S4. Time trends in registered psychiatric diagnosis, prescription claims of psychiatric medication and/or receipt of psychological therapy among women in the Stockholm Region 2007–2017 per age group. Figure S5. Time trends in registered psychiatric diagnosis, prescription claims of psychiatric medication and/or receipt of psychological therapy among men in the Stockholm Region 2007–2017 per age group. [file 12888_2020_2749_MOESM1_ESM.docx]

# Supplemental Appendix

Trends in psychiatric diagnoses, medications and psychological therapies in a large Swedish region: a population-based study. Forslund T, Kosidou K, Wicks S, Dalman C.

Corresponding author: Tomas Forslund, Karolinska Institutet/Stockholm Region, tomas.forslund@sll.se

Supplemental Table 1 – ICD-code definitions

| **Psychiatric diagnosis** | **ICD-code beginning with** |
| --- | --- |
| Depression | F32, F33, F34, F38, F39 |
| Anxiety disorders | F40, F41, F42, F43.1 |
| Reaction to severe stress, and adjustment disorders | F43 excluding F43.1 |
| Psychotic disorder | F20-F29 |
| Bipolar disorder | F30, F31 |
| Eating disorders | F50 |
| ADHD | F90 |
| Pervasive developmental disorders | F84 |
| Burnout syndrome | F438A |

Supplemental Table 2 – ATC-code definitions

| **Psychiatric medication** | **ATC-code beginning with** |
| --- | --- |
| Antidepressants | N06A (excluding N06AA09) |
| Antipsychotics | N05A |
| Psychostimulants | N06B |

**Supplemental Figure 1 - Age and sex standardized time trends for mental health care utilization in primary and secondary care respectively or both in the Stockholm Region (1 758 337 adult individuals in 2017)**

**Supplemental Figure 2 - Age and sex standardized prescription claims of psychiatric medication (antidepressants, antipsychotics and psychostimulants) among adults in the Stockholm Region 2011-2017 (1 758 337 adult individuals in 2017)**

**Supplemental Figure 3 - Adult individuals with a diagnosis of a psychiatric disorder during 2013-2017 by level of care in the Stockholm Region (population 1 758 337 individuals)**

**Supplemental Figure 4 - Time trends in registered psychiatric diagnosis, prescription claims of psychiatric medication and/or receipt of psychological therapy among women in the Stockholm Region 2007-2017 per age group**

**Supplemental Figure 5 - Time trends in registered psychiatric diagnosis, prescription claims of psychiatric medication and/or receipt of psychological therapy among men in the Stockholm Region 2007-2017 per age group**
